# Selective Constraints in Experimentally Defined Primate Regulatory Regions

**DOI:** 10.1371/journal.pgen.1000157

**Published:** 2008-08-15

**Authors:** Daniel J. Gaffney, Ran Blekhman, Jacek Majewski

**Affiliations:** 1 McGill University, Montréal, Québec, Canada; 2 Genome Québec Innovation Centre, Montréal, Québec, Canada; 3 Department of Human Genetics, University of Chicago, Chicago, Illinois, United States of America; Wellcome Trust Sanger Institute, United Kingdom

## Abstract

Changes in gene regulation may be important in evolution. However, the evolutionary properties of regulatory mutations are currently poorly understood. This is partly the result of an incomplete annotation of functional regulatory DNA in many species. For example, transcription factor binding sites (TFBSs), a major component of eukaryotic regulatory architecture, are typically short, degenerate, and therefore difficult to differentiate from randomly occurring, nonfunctional sequences. Furthermore, although sites such as TFBSs can be computationally predicted using evolutionary conservation as a criterion, estimates of the true level of selective constraint (defined as the fraction of strongly deleterious mutations occurring at a locus) in regulatory regions will, by definition, be upwardly biased in datasets that are *a priori* evolutionarily conserved. Here we investigate the fitness effects of regulatory mutations using two complementary datasets of human TFBSs that are likely to be relatively free of ascertainment bias with respect to evolutionary conservation but, importantly, are supported by experimental data. The first is a collection of almost >2,100 human TFBSs drawn from the literature in the TRANSFAC database, and the second is derived from several recent high-throughput chromatin immunoprecipitation coupled with genomic microarray (ChIP-chip) analyses. We also define a set of putative *cis*-regulatory modules (pCRMs) by spatially clustering multiple TFBSs that regulate the same gene. We find that a relatively high proportion (∼37%) of mutations at TFBSs are strongly deleterious, similar to that at a 2-fold degenerate protein-coding site. However, constraint is significantly reduced in human and chimpanzee pCRMS and ChIP-chip sequences, relative to macaques. We estimate that the fraction of regulatory mutations that have been driven to fixation by positive selection in humans is not significantly different from zero. We also find that the level of selective constraint in our TFBSs, pCRMs, and ChIP-chip sequences is negatively correlated with the expression breadth of the regulated gene, whereas the opposite relationship holds at that gene's nonsynonymous and synonymous sites. Finally, we find that the rate of protein evolution in a transcription factor appears to be positively correlated with the breadth of expression of the gene it regulates. Our study suggests that strongly deleterious regulatory mutations are considerably more likely (1.6-fold) to occur in tissue-specific than in housekeeping genes, implying that there is a fitness cost to increasing “complexity” of gene expression.

## Author Summary

Changes in gene expression have been suggested to play a major role in mammalian evolution. In eukaryotes, gene expression is primarily controlled by sites, such as transcription factor binding sites (TFBSs), located in the noncoding region of the genome. The majority of these TFBSs remain unannotated, however, because they are typically short, degenerate, and laborious to identify experimentally. As a result, the effects of mutations in TFBSs on organism fitness remain poorly understood. We collected a dataset of TFBSs derived from the experimental biology literature and recent high-throughput studies to estimate the proportions of new mutations in TFBSs that have strongly deleterious and strongly beneficial effects upon organism fitness. We find that a relatively high proportion of new mutations in TFBSs are strongly deleterious, although it appears that relatively few are adaptive. We also demonstrate that the fraction of strongly deleterious regulatory mutations is correlated with the breadth of expression of the regulated gene. Thus, ubiquitously expressed genes are likely to experience fewer deleterious regulatory mutations than those expressed in a small number of tissues.

## Introduction

Changes in gene regulation are likely to play an important role in evolution [1],[2]. However, compared to protein-coding sequences, the fitness effects of regulatory mutations remain poorly understood. Furthermore, the relationship between changes in gene regulatory regions and the expression phenotype of the regulated gene are unclear. Both of these issues are partly a result of poor annotation of the sites that control gene regulation, the vast majority of which are likely to be noncoding. For example, transcription factor binding sites (TFBSs), a major component of regulatory architecture, are small (6–15 bp), laborious to identify experimentally and potentially degenerate. Furthermore, due to their small size, genuine TFBS are difficult to differentiate from similar, randomly-occurring sequences that are present in large numbers in mammalian genomes.

In an attempt to address the problem of annotation, evolutionary conservation has become popular as a metric for identifying putative regulatory regions [3]. However estimates of the true level of selective constraint (defined as the proportion of mutations which are strongly deleterious) in regulatory DNA will, by definition, be biased upwards in datasets predicted using evolutionary conservation as a criterion.

One way to address this problem is to focus solely on regulatory regions which have been defined primarily by experimental rather than evolutionary criteria. In this study, we estimated levels of selective constraint in mammalian regulatory noncoding DNA using two complementary datasets, both of which draw upon experimental data. The first was derived from the literature collected in the TRANSFAC database [4], such that every TFBS is supported by at least a single refereed publication. The advantages of this dataset are twofold. First, our dataset consists of individual TFBSs for which experimental support exists and which, according to an analysis by publication date (see Discussion), appear to be subject to relatively little ascertainment bias with respect to evolutionary conservation. Second, the literature in TRANSFAC also provides substantial information on the gene regulated and the transcription factor bound for each TFBS. Thus, the TFBSs in our dataset can be assigned to a specific gene reliably, and we can also determine at least some of the transcription factors (TFs) which regulate a specific gene's expression. Our second dataset comprises sequences which have been identified as potentially transcription-factor-binding using chromatin immunoprecipitation combined with genomic microarray (ChIP-chip) analyses. Specifically, we combine the sequences annotated in refs 5–11. While the resolution at which regulatory sites are identified is undoubtedly lower in the ChIP-chip dataset than in our TFBS dataset, our ChIP-chip sequences will still be highly enriched for functional regulatory DNA.

Using these two datasets, we addressed the following questions: (i) what fraction of regulatory mutations in primates are strongly deleterious, (ii) does the fraction of strongly deleterious mutations at TFBSs vary between primates, (iii) what fraction of substitutions in human regulatory regions have been driven to fixation by positive selection, (iv) how does the selective constraint of human regulatory noncoding regions relate to the expression profile of the gene they regulate and (v) does the rate of protein evolution of a TFs also relate to the expression profile of the regulated gene?

## Results

Of the 2494 human TFBS accessions in TRANSFAC 10.2 we were able to successfully locate a total of 2097 TFBSs on human genome assembly 18. Many of these regions overlapped and so we were able to define a total of 1508 unique TFBSs, corresponding to over 18 kb of sequence in the human lineage. The TFBSs in our study were a highly heterogeneous mix of noncoding binding regions which included TATA boxes, CCAAT/enhancer binding protein sites (C/EBP sites), cAMP response elements (CREs), interferon-stimulated response elements (ISREs) as well as binding sites for a variety of common TFs such as cMyc, Sp1, NF-κB, CREB and p53. Those 1355 TFBSs for which we were able to extract orthologous sequence from both the chimpanzee and macaque genomes were grouped according to location into a total of 468 case regions, as described in the Methods.

**Figure 1:**
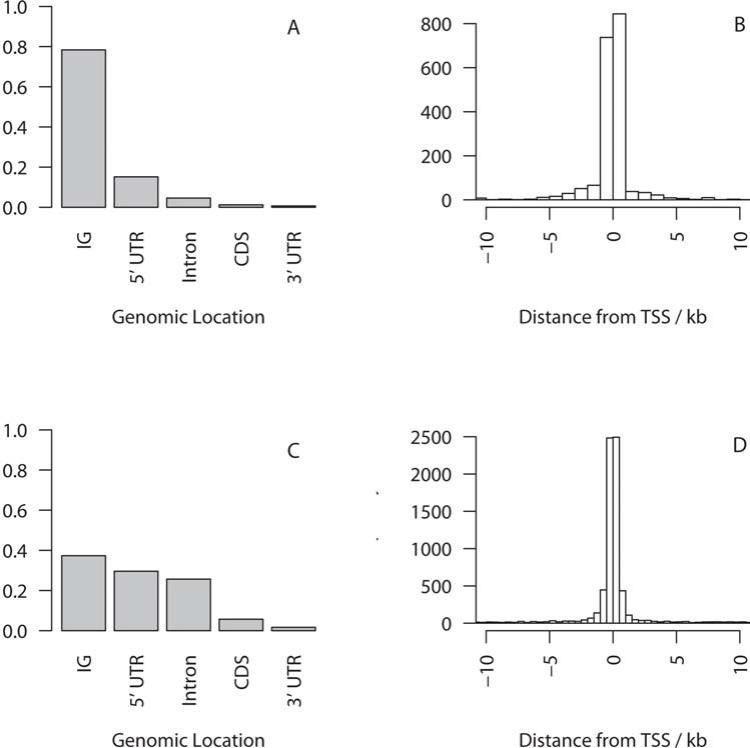
Proportion of the total number of sites contributed by TFBSs (A) and ChIP-chip sequences (C) in different genomic regions, and frequency distribution of the distance of TFBS (B) or ChIP-chip sequence (D) from the transcription start site (TSS) of the regulated gene.

Of the 12364 ChIP-chip sequences annotated in refs 5–11 we defined 10104 unique, nonoverlapping regions corresponding to 5.3Mb of human sequence which were divided into 6712 “case” regions. The genomic locations of the TFBSs and ChIP-chip sequences are shown in Figure 1.

Using parsimony, we estimated rates of nucleotide substitution at non-CpG-prone sites in the TFBSs, their flanking sequences, the ChIP-chip sequences and our neutral control regions. Both TFBSs and ChIP-chip sequences are evolving significantly slower than their respective controls (Figure S1). This suggests that our controls contain significantly more neutrally evolving sites than both our regions of interest and likely provide a reasonable estimate of the local mutation rate, a major assumption of our method.

**Figure 2:**
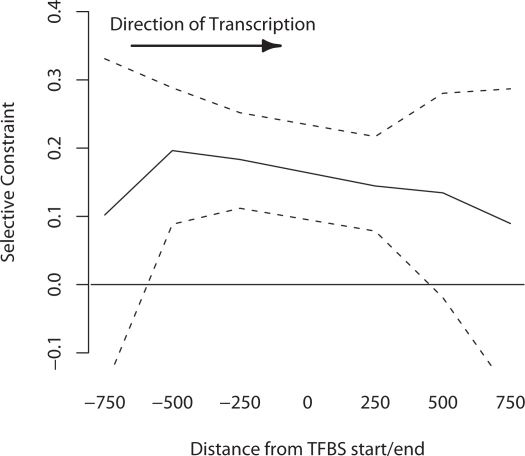
Selective constraint of flanking sequence located between two annotated TFBSs that are <1.5 kb apart. Dotted lines show 95% confidence intervals estimated by bootstrapping the data by case-control region, 1000 times.

**Figure 3:**
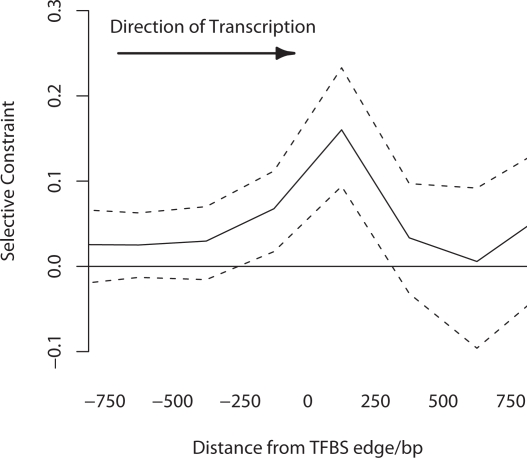
Selective constraint of flanking sequences for which the annotated TFBS was >1.5 kb from another annotated TFBS, coding sequence or TSS. Dotted lines show 95% confidence intervals estimated by bootstrapping the data by case-control region, 1000 times.

Individual TFBSs are highly non-randomly spatially distributed in metazoans, and are typically located within clusters of other binding sites termed *cis*-regulatory modules (CRMs). These clusters serve to bind multiple TFs whose combined action controls the level and location of gene expression. We investigated whether this modular organisation of regulatory DNA was reflected by the level of selective constraint in the noncoding DNA surrounding known TFBSs. In order to distinguish between regions within CRMs versus those at the outermost edge we divided our dataset of flanking sequences into two groups: (i) those flanking regions that lie between two annotated TRANSFAC binding sites which are less than 1.5 kb apart and (ii) those flanking regions that are greater than 1.5 kb from another annotated binding site, coding sequence or TSS. These criteria will enrich group 1 in sequence lying within a CRM, while those in group 2 will be enriched in sequence lying at the edge of a CRM. It is clear from Figures 2 and 3 that sequence adjacent to our TFBSs is selectively constrained for a considerable distance upstream and downstream of the binding site itself, suggesting that our annotated TFBSs are likely surrounded by other regulatory regions. Furthermore many of the sites which, according to their TRANSFAC annotation, co-regulate the same gene, are located in close proximity indicating that they function co-cooperatively as a CRM. We therefore defined a set of 696 putative CRMs (pCRMs) in our dataset as all TFBSs which regulated the same gene and were <350 bp from one another, their intervening sequence and up to 350 bp of flanking sequence. The mean length of a pCRM in our data was 415 bp.

### Constraint in Primate TFBSs, pCRMs, and ChIP-chip Sequences

**Figure 4:**
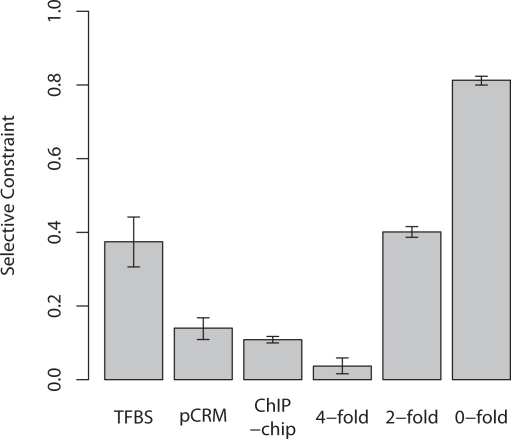
Estimates of selective constraint at TFBSs, pCRMs and ChIP-chip sequences averaged across all three primates and 4-fold, 2-fold and 0-fold degenerate sites. Genes used were those regulated by the TFBSs and inferred to be regulated by the ChIP-chip sequences.

We next estimated the level of selective constraint at TFBSs, pCRMs and in ChIP-chip sequences (Figure 4). TFBSs appear to be reasonably highly constrained, approximately equivalent to a 2-fold degenerate synonymous site. This result is in good agreement with previous studies which have suggested that a reasonable proportion of TRANSFAC binding sites are conserved between human and a variety of mammalian species [12]–[16]. Estimates of constraint in our putative *cis*-regulatory modules and sequences annotated by ChIP-chip experiments are somewhat similar (0.14 and 0.11, respectively) suggesting that ChIP-chip studies can serve as reliable guides to functional regulatory regions in humans when compared with more traditional methods of identification.

### Variation in Selective Constraint between Primates

**Figure 5:**
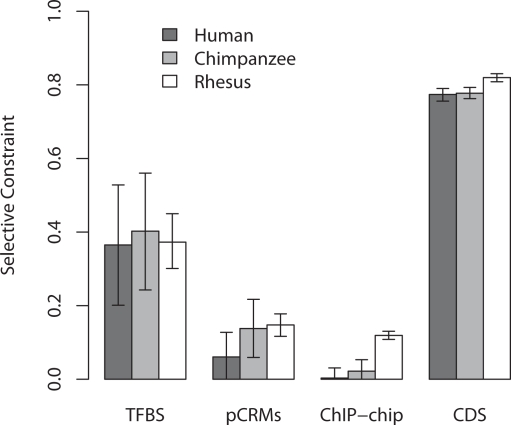
Selective constraint of regulatory noncoding (TFBSs, pCRMs and ChIP-chip sequences) and coding (nonsynonymous sites) DNA in humans, chimpanzees and macaques.

It has been suggested that regulatory DNA in primates is under relaxed selective constraint relative to rodents [17]. This has been attributed to the reduction of effective population size in primates facilitating the fixation of slightly deleterious mutations in gene control regions. Effective population size is likely to vary between humans, chimpanzees and macaques and we therefore investigated whether any significant difference in constraint of regulatory noncoding regions existed between humans and their close relatives. It is clear from Figure 5 that selective constraints in regulatory noncoding DNA vary significantly between primate species (1-way ANOVA, P<10^−16)^, and that this is primarily a result of a reduction in constraint in hominins (*post-hoc* Tukey test, P<10^−5^ human vs rhesus, chimp vs rhesus) when compared with rhesus macaques. Summing over both coding and noncoding sites, we also find that mean selective constraint is also reduced somewhat in humans, compared with chimpanzees (0.254 versus 0.279), although this difference is only marginally significant (Bootstrap t-test, P<0.07). Two possible explanations for the reduction in constraint are that humans and chimpanzees have accumulated substantially greater numbers of deleterious mutations or are experiencing higher rates of adaptive evolution in their regulatory regions.

### Adaptive Evolution in Primate Regulatory DNA

**Figure 6:**
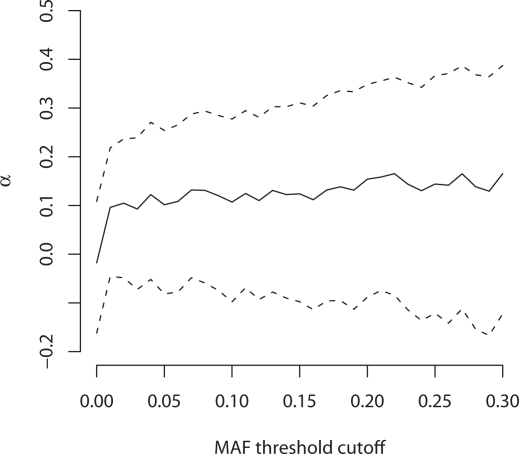
Fraction of adaptive substitutions (*α*) in primate pCRMs and ChIP-chip sequences versus the threshold minor allele frequency (MAF) that was excluded from the analysis prior to the estimation of *α* (see text). Confidence intervals are shown as dashed lines and were estimated by bootstrapping the data by case-control region, 10000 times.

In order to investigate whether the reduced constraints we observed in human regulatory noncoding DNA were the result of adaptive evolution we estimated the proportion of substitutions (α) which were driven to fixation by positive selection in our pCRMs and ChIP-chip sequences using the McDonald-Kreitman framework [18],[19]. We were able to map 232 of our pCRMs and ChIP-chip sequences onto regions sequenced by the NIEHS Environmental Genome Project (EGP; http://egp.gs.washington.edu). Polymorphism data was taken from the EGP as this dataset is free of ascertainment bias, relative to other large polymorphism datasets, such as HapMap [20]. McDonald-Kreitman analyses assumes that all mutations can be divided into strongly selected (positively or negatively) or strictly neutral classes. One problem with this is that a non-negligible fraction of new mutations in species with small effective population sizes, such as primates, may be weakly negatively selected. To account for this possibility we estimated α using both all segregating sites and excluding those sites where the minor allele frequency MAF ranged from 0.01 to 0.30, many of which are likely to be slightly deleterious [21]. We find no evidence of adaptive evolution in human regulatory regions and our estimate of α is not significantly different from zero across the entire range of excluded, low frequency polymorphisms (Figure 6).

### Selective Constraint in Regulatory Noncoding DNA and Gene Expression Profile

**Figure 7:**
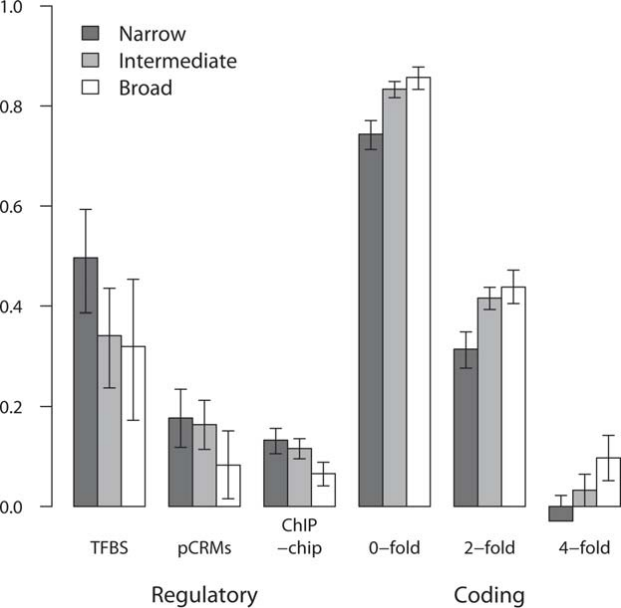
Constraint in regulatory noncoding DNA (TFBSs, pCRMs, ChIP-chip sequences) and coding regions (nonsynonymous, 2-fold and 4-fold synonymous sites) versus gene expression breadth. Narrow, intermediate and broad expression breadth were defined using lower (2 tissues) and upper quartiles (>30 tissues) of the distribution of number of tissues expressed per gene. Constraint at pCRMs, ChIP-chip sequences, nonsynonymous, 2-fold and 4-fold degenerate sites was significantly correlated with number of tissues in which a gene was expressed (Pearson r = −0.144, P<0.005; r = −0.092,<5.07×10^−7^; r = 0.176, P<1.22×10^−12^; r = 0.099, P<6.63×10^−5^; r = 0.110, P<9.36×10^−6^, respectively).

We next investigated whether constraint in our regulatory sites covaried with the expression breadth of the gene regulated. Expression profile of the genes inferred to be regulated by our pCRMs and ChIP-chip sequences was estimated from the human microarray data of Su *et al* [22]. A gene was defined as expressed in a specific tissue based on the Affymetrix MAS5 presence/absence calls. Our results were qualitatively unchanged when gene expression was designated using a cutoff probe intensity value (data not shown). For comparison, we also estimated constraint at the nonsynonymous (0-fold degenerate) and synonymous sites of the genes adjacent to our regulatory regions. The results of this analysis are presented in Figure 7. There is a clear relationship between selective constraint in regulatory regions and breadth of expression of the regulated gene. pCRM and ChIP-chip selective constraint is significantly negatively correlated with the number of tissues in which a gene is expressed (P<0.005, P<5.07×10^−7^, respectively). This is not a function of the number of annotated TFBSs in our pCRMs, which is uncorrelated (Pearson r = 0.015;P<0.738) with pCRM constraint. A similar relationship appears to exist between TFBS selective constraint and expression breadth. In particular, the TFBSs of tissue-specific genes are more highly constrained than those of intermediate and broadly expressed genes (2-sided t-test; P<0.012). However, the equivalent regression is not significant at least in part due to the high error involved in estimating selective constraint from a small number of sites between closely related species. The relationship between constraint and expression profile is reversed in protein-coding sequence where constraint increases with increasing expression breadth (P<1.11×10^−15^ and P<1.70×10^−6^, nonsynonymous and 2-fold degenerate, respectively), a result supported by previous work [23],[24],[25]. Interestingly, constraint at 4-fold degenerate synonymous sites is also positively correlated with expression breadth (P<2.60×10^−6^), suggesting that constraints on mRNA stability and/or splicing efficiency reflect those on protein structure, with respect to expression breadth. These results are not a product of different rates of nucleotide substitution in the intronic control regions of genes with differing expression breadth; we find that divergence in all controls used in our study was uncorrelated with breadth of expression of the gene in which they reside (Pearson r = −0.004 P>0.83; Figure S2).

It has previously been shown that mammalian promoters can be divided into two classes, CpG-rich and CpG-poor, based on the distribution of %CpG in human promoter regions [26] and these two classes of promoter region are associated with expression breadth. Following ref 26, we divided our pCRM and ChIP-chip sequences into CpG-rich and CpG-poor classes, to investigate whether this could explain the relationship we find between expression breadth and conservation. The majority (95%) of our pCRMs and ChIP-chip sequences are CpG-rich by the definition in ref 26 i.e. they have a normalized CpG content of >0.35. Within this CpG-rich class, constraint of regulatory regions is still significantly negatively correlated with expression breadth (Pearson r = −0.103, P<3.68×10^−8^). We also tested the influence of CpG content by regressing pCRM and ChIP-chip constraint on their %CpG. The slope of this regression is negative and significantly different from zero (simple linear regression b = −0.058, P<0.028). However, the residuals of this regression are still negatively correlated with expression breadth (Pearson r = −0.086, P<5.99×10^−7^). These results suggest that, while CpG content is indeed correlated with constraint of regulatory DNA this does not explain the majority of the relationship we see between regulatory constraint and expression profile.

### Transcription Factor Dn/Ds and Gene Expression Profile

**Figure 8:**
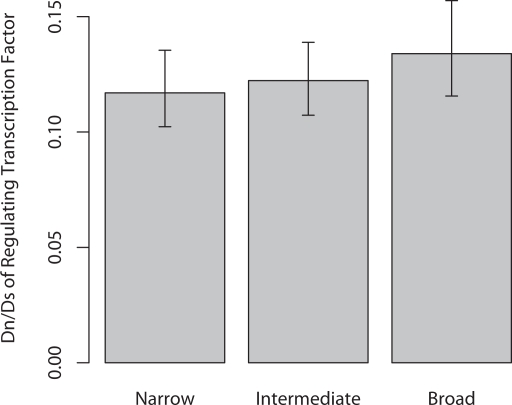
Dn/Ds of the transcription factors (TFs) regulating gene expression versus gene expression breadth. Dn/Ds was estimated from human-macaque alignments, treating all TFs known to regulate each gene as a single sequence.

One advantage of our TFBS dataset is that we can identify which TF(s) control the expression of a specific gene, and that this relationship is also supported by experimental evidence. We therefore investigated whether the rate of protein evolution (estimated as Dn/Ds, the ratio of nonsynonymous to synonymous substitution) in a TF bore any relationship to the expression breadth of the regulated gene. Dn/Ds was estimated summing over all sites of all TFs which were known to regulate a specific gene. We obtained Dn/Ds estimates for 185 TFs which regulate 349 genes. The results of this analysis are presented in Figure 8. Interestingly we find that TF Dn/Ds ratio is significantly positively correlated with gene expression breadth (Pearson r = 0.15;P<0.005). We tested whether this result was an artifact of summing across multiple TFs by restricting our analysis to the 99 genes which were regulated by a single TF. Despite this reduced dataset TF Dn/Ds is still marginally significantly correlated with gene expression breadth (P<0.076).

One major factor which influences the rate of protein evolution is their structure. We therefore tested whether the relationship between transcription factor Dn/Ds and gene expression profile was influenced by the transcription factor structural class. We divided the regulating TFs into four protein “superclasses” based on the transcription factor protein classification tree in TRANSFAC. Of our 185 TFs we were able to assign 141 to either leucine zipper factors (LZ; 27 TFs), zinc-coordinating DNA-binding domains (ZC; 50 TFs), helix-turn-helix proteins (HTH; 42 TFs) and *β*-scaffold factors with minor groove contacts (BSF; 22 TFs). As expected, we find clear differences in the Dn/Ds ratio of each the four classes (1-way ANOVA P≪1.69×10^−5^; Figure S3). However, we find no relationship between protein structural class and expression breadth of the regulated gene (1-way ANOVA P<0.464; Figure S4). This suggests that the relationship we observe between the rate of protein evolution in a TF and the expression breadth profile of the regulated gene are independent of the protein structure of the TF.

## Discussion

We have presented a study of the fitness effects of mutations in primate regulatory noncoding DNA. The regulatory regions included in our study are supported by a variety of experimental sources, both based on the extensive experimental biology literature, and inferred from more recent, high-throughput studies. Our study confirms that experimentally validated regulatory noncoding regions are selectively constrained, a result supported by other previous studies of datasets of TRANSFAC TFBSs in mammals [12]–[16]. Our estimates imply that ∼37% of new spontaneous mutations in primate TFBSs have a strongly deleterious effect and are removed by purifying selection. We find that the proportion of strongly deleterious noncoding regulatory mutations varies significantly even between closely-related primate species, reflecting a similar trend in coding DNA. We find no evidence for adaptive evolution in human regulatory regions, suggesting that these differences in selective constraint between primate taxa are likely to primarily reflect variations in effective population size. Our study also clearly shows that the level of selective constraint in primate regulatory DNA depends upon the expression profile of the gene regulated. Intriguingly, we also find higher constraint in the regulatory regions of tissue-specific genes is reflected in the rate of protein evolution of the TFs that interact with them.

Our study suggests that at least some fraction of human regulatory DNA is accumulating slightly deleterious mutations at an accelerated rate relative to other, closely-related primate species. We find no evidence of adaptive evolution in our regulatory regions. Nonetheless, a number of recent reports have suggested accelerated evolution in human noncoding DNA [27],[28],[29]. There may be a number of reasons that we do not observe such an effect. Firstly, we restrict our analysis to experimentally-supported regulatory noncoding DNA and exclude CpG prone sites entirely from our analysis and may therefore lack sufficient power to detect all but very strong selection. Secondly our analysis is based upon the McDonald-Kreitman test which assumes that all adaptive mutations are strongly selected. However, recent work has suggested that at least some fraction of adaptive mutations may be weakly selected [30]. Although our degree of confidence in our estimates of *α* is small, the increasing numbers of high quality ChIP-chip datasets combined with larger resequencing studies will improve the accuracy of estimates of this important parameter.

The results we have presented also shed light on the relationship between gene expression and selective constraint of both the TF and TFBSs which ultimately control this expression. A straightforward interpretation of our results is that selective constraint of regulatory DNA parallels the “complexity” of expression of the gene it regulates i.e. genes that are required to be “switched on” ubiquitously have a simpler, more degenerate regulatory architecture than those genes which require delicate control of the location and timing of expression. This interpretation is supported by a recent study of human-mouse promoter regions [31]. Furthermore, this hypothesis is intuitively appealing when we consider that tissue-specific genes may require regulatory sites both to up-regulate expression in the correct tissue, but also to suppress expression in an inappropriate tissue, a function that is presumably absent from the regulatory region of a broadly-expressed gene. Taken together with estimates of constraint in protein-coding sequence our study suggests the following: broadly expressed genes produce a protein whose structure is tightly maintained by purifying selection but whose regulatory architecture is degenerate. Tissue-specific genes on the other hand require a more elaborate and specific regulatory apparatus, but the protein produced by such genes is less rigorously maintained by selection. It has been suggested that mutations affecting the regulation of tissue-specific genes are less likely to be strongly deleterious than those in broadly-expressed genes, given that they are expressed in a subset of tissues [32]. However, our results support the opposite interpretation.

Although the correlations we observe between regulatory constraint and expression breadth are weak, we note that the experimental methods of annotation of regulatory sites are imperfect, and the numbers of sites which we have used in this study are relatively small, by genomic standards. In addition, estimates of selective constraint are essentially a ratio of ratios, making them inherently noisy. In the light of this, the strength of our correlations is perhaps less surprising. It is also likely that our results to a certain extent reflect the variation in constraint of the noncoding DNA surrounding different functional “classes” of genes, as demonstrated previously (e.g. ref 33). We note, however, that the relationship between gene expression profile and gene functional class as assigned by ontological classification is uncertain. In addition, without a complete annotation of functional noncoding sites, we cannot distinguish whether between-gene variation in constraint of surrounding noncoding regions reflects variation in the *number* of constrained sites or in the *intensity of purifying selection* at these sites. One advantage of the approach we have employed here is that we can at least partially disentangle these two factors; our results suggest that the intensity of purifying selection at primate TFBSs is indeed greater in tissue-specific genes (Figure 7).

We note that our estimates of constraint may also be biased upwards for two reasons. The UCSC whole genome alignments are assembled with reference to the human genome and it is therefore possible that their use in our study could exclude weakly conserved unalignable TFBSs. This could potentially lead to an overestimate of the true level of constraint. However, we suggest that the impact of this is likely to be small given that such a bias will affect our control regions also, and thus will cancel in the estimation of constraint. In addition, although ascertainment bias in the TRANSFAC annotations is reduced, compared to some computationally-predicted regions, it is unlikely to be zero, as phylogenetic footprinting has become more frequently used over time as a means of selecting candidate regulatory regions for experimental testing. Unfortunately it is difficult to quantify this bias. However, if phylogenetic footprinting has had a significant effect upon our TFBS dataset we might predict that, on average, those TFBSs that were annotated relatively recently would be less diverged than those annotated in the more distant past, given the dramatic increase in the use of comparative genomics in recent years. We find that divergence is not significantly correlated with year of appearence of the supporting publication (Figure S5). We do find that TFBSs published before 1996 (the median age of publication of human TRANSFAC TFBSs) are marginally (∼8%) more diverged than those published during or after 1996, although this difference is not significant (Bootstrap t-test, P<0.19). Thus, although our estimates of TFBS constraint may be upwardly biased, this bias is likely to be small.

One straightforward implication of our results is that deleterious regulatory mutations are more likely to disrupt genes with tissue-specific expression, as a result of higher levels of constraint in both their regulatory regions and the protein-coding sequence of the TFs that bind to these regions. We estimate that deleterious mutations will occur on average 1.6-fold more often in regulatory regions of tissue-specific (≤3 tissues) than housekeeping genes (>35 tissues). This conclusion has interesting implications when we consider recent evidence suggesting that there are substantially more tissue-specific genes in primates compared with rodents [34]. Our data imply that the penalty for an increase in expression “complexity” is a concurrent increase in the genomic deleterious mutation rate. This penalty may, however, be offset by a corresponding decrease in the proportion of deleterious protein-coding mutations.

## Materials and Methods

### Data Collection

The data used in this study were collected from two sources. We first used the literature in TRANSFAC release 10.2 [4] to compile a dataset of known, experimentally-supported TFBSs. For those TFBSs which were linked to a specific EMBL accession, we BLASTed the binding site and up to 400 bp of flanking sequence against the human genome (assembly 18). Query sequences which matched a single, unique region in the human genome with a BLAST *e*-value of <10^−5^ were accepted. Those regions which matched more than a single location were resolved manually by comparison with any existing annotation in TRANSFAC, or excluded. For those TFBSs which were not linked to an existing EMBL record, we BLASTed the binding site sequence against the transcript of the RefSeq gene regulated, as recorded in TRANSFAC, with 20 kb flanking sequence. We accepted any binding site which matched a single unique location in this sequence, with <99% identity, for the full length of the binding site. We hereafter refer to these data as “TFBS” sequences. All binding sites were checked to be in the appropriate chromosomal location with respect to the gene they regulate. Our second dataset was derived from DNA sequences bound by a variety of TFs in 7 recent chromatin immunoprecipitation-coupled DNA microarray (ChIP-on-chip) analyses [5]–[11]. The locations of these sequences were extracted from the “fragment” table of TRANSFAC 10.2 and updated to the latest assembly of the human genome. We hereafter refer to these data as “ChIP-chip” sequences.

To estimate the level of selective constraint, we needed to compare substitution rates in our TFBS and ChIP-chip datasets with those in an appropriate neutrally-evolving control region, which has a mutation rate equal to that of the region of interest. Previous analyses [33],[35] have suggested that, in mammals, intronic regions outside the first intron and the splice sites are the fastest evolving in the genome and among the best candidates for neutrally-evolving sequence. Because sites in both datasets were highly nonrandomly distributed across the genome, we sought to define a single control region for a “case” region of binding sites, rather than for each individual annotated sequence. A “case” was defined as a group of TFBSs or ChIP-chip sequences in which the maximum distance between each cluster member and its nearest neighbour was 100 kb. A control region for each “case” was defined as the window which extended up to 250 kb either side of the midpoint of cluster. All non-first intronic sequence, excluding the first and last 100 bp, within this 500 kb window were denoted as control regions for the “case” region. Given that mutation rates in mammals appear to vary across megabase scales [36],[37] it is likely that the mutation rate in our control sites will not differ significantly from that in our “case” sites. In a minority of cases (<5% of TFBSs and ChIP-chip sequences), suitable intronic controls were unavailable. In this case, we used nearby intergenic sequence which was greater than 1 kb from an annotated coding sequence. All exon locations were taken from RefSeq annotations. The selection of an arbitrary between-site distance of 100 kb allowed us to define 473 unique, nonoverlapping binding site “cases”, each with a unique set of intronic controls. Likewise, we defined 6712 ChIP-chip “cases” from our 10104 unique ChIP-chip regions.

### Analysis

For all TFBSs, ChIP-chip sequences and their corresponding control regions, aligned sequence data from the human, chimpanzee (assembly 2) and macaque (assembly 2) genomes was extracted from the 28-way vertebrate alignments available in the UCSC genome browser database [38]. In order to minimize the effects of poor sequence quality in the chimp and macaque genomes we masked all sites which were assigned a base quality of less than 20 in either species. Lineage-specific substitution rates were estimated using parsimony. Estimates of substitution rates were not corrected for multiple hits, given that this will make little difference between closely related species.

In all cases, selective constraint, C, was estimated as:
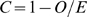
where O is the number of substitutions observed in the TFBS or other region of interest and E is the number of substitutions expected under neutral evolution:
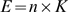
where n is the length of the TFBS or other region of interest and K is the substitution rate estimated from the control region. Unless stated otherwise, all confidence intervals were estimated by bootstrapping the data by binding site “case”, 1000 times.

The method of estimation of selective constraint employed here explicitly accounts for local mutational variation. Previous studies of experimentally validated mammalian regulatory DNA (e.g. refs 14–16), have not accounted for such variation. This is particularly important in our study for two reasons. Firstly, substantial within-genome mutational variation is known to occur in mammals [37],[39] meaning that the expectation of conservation under neutrality will vary from one genomic region to the next. This can substantially impact estimates of conservation between very closely related species, such as humans and chimpanzees. Secondly, regulatory regions frequently reside in CpG islands, where the level of CpG hypermutability is known to differ from other, more heavily methylated regions of the genome. Given that CpG mutations make up a disproportionately large number of all mutations in mammals, it is important to correct for variations in the level of CpG hypermutability to avoid overestimating constraint in regions of lowered CpG hypermutability such as CpG islands. Here, we account for variation in the frequency and mutability of CpG dinucleotides by excluding non CpG-prone sites (not preceded by ‘C’ or followed by ‘G’).

TF Dn/Ds ratios were estimated from human-macaque alignments in the Cornell orthologues dataset using PAML [40]. In the case where multiple factors were known to regulate a gene *x*, Dn/Ds (*ω_x_*) was estimated summing over all TFs, T_x_  =  *t*_1_,…,*t_n_* as:
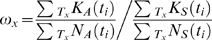
where *K_A_(t_i_)* and *K_s_(t_i_)* are the number of pairwise nonsynonymous and synonymous substitutions in TF*^i^*, and *N_A_(t_i_)* and *N_s_(t_i_)* are the number of pairwise nonsynonymous and synonymous sites in TF*i*, respectively.

## Supporting Information

Figure S1 Nucleotide substitution rates at TFBSs, ChIP-chip sequences and their respective neutral control regions summed across all three species.
(0.43 MB EPS)

Figure S2 Relationship between divergence (estimated summing across all three species in our study) in our neutral control regions and the expression breadth of gene in which they reside. Control divergence is not significantly correlated with expression breadth (P<0.83).
(1.54 MB EPS)

Figure S3 Boxplots of TF Dn/Ds ratio by structural superclass. BSF: β-scaffold factors with minor groove contacts; HTH:helix-turn-helix proteins; LZ:leucine zipper factors; ZC: zinc-coordinating DNA-binding domains.
(0.44 MB EPS)

Figure S4 Boxplots of expression breadth of gene by structural superclass of the regulating TF. BSF: β-scaffold factors with minor groove contacts; HTH:helix-turn-helix proteins; LZ:leucine zipper factors;ZC: zinc-coordinating DNA-binding domains.
(0.48 MB EPS)

Figure S5 TFBS divergence summed over all species plotted against year of publication of supporting literature.
(0.97 MB EPS)
